# Robust design of a machine learning-based GNSS NLOS detector with multi-frequency features

**DOI:** 10.3389/frobt.2023.1171255

**Published:** 2023-07-28

**Authors:** Omar García Crespillo, Juan Carlos Ruiz-Sicilia, Ana Kliman, Juliette Marais

**Affiliations:** ^1^ Navigation Department, Institute of Communication and Navigation, German Aerospace Center (DLR), Oberpfaffenhofen, Germany; ^2^ Univ Gustave Eiffel, COSYS-LEOST, Villeneuve d’Ascq, France

**Keywords:** global navigation satellite system, non-line-of-sight propagation, machine learning, urban environment, local threats

## Abstract

The robust detection of GNSS non-line-of-sight (NLOS) signals is of vital importance for land- and close-to-land-based safe navigation applications. The usage of GNSS measurements affected by NLOS can lead to large unbounded positioning errors and loss of safety. Due to the complex signal conditions in urban environments, the use of machine learning or artificial intelligence techniques and algorithms has recently been identified as potential tools to classify GNSS LOS/NLOS signals. The design of machine learning algorithms with GNSS features is an emerging field of research that must, however, be tackled carefully to avoid biased estimation results and to guarantee algorithms that can be generalized for different scenarios, receivers, antennas, and their specific installations and configurations. This work first provides new options to guarantee a proper generalization of trained algorithms by means of a pre-normalization of features with models extracted in open-sky (nominal) scenarios. The second main contribution focuses on designing a branched (or parallel) machine learning process to handle the intermittent presence of GNSS features in certain frequencies. This allows to exploit measurements in all available frequencies as compared to current approaches in the literature based on only the single frequency. The detection by means of logistic regression not only provides a binary LOS/NLOS decision but also an associated probability which can be used in the future as a means to weight-specific measurements. The detection with the proposed branched logistic regression with pre-normalized multi-frequency features has shown better results than the state-of-the-art algorithms, reaching 90% detection accuracy in the validation scenarios evaluated.

## 1 Introduction

Global navigation satellite systems (GNSSs) are widely used in transportation applications to localize and navigate vehicles. Compared to aviation, land and close-to-land applications suffer from an additional challenge to GNSS positioning: the presence of multiple local threats. These include, among others, multipath, non-line-of-sight (NLOS) signal reception, and interference. Because of these threats, the implementation of GNSS for safety-related applications is still restricted almost exclusively to aviation applications. Other means of transport like railway and emerging applications like autonomous vehicles and urban air mobility will also need to ensure the reliability and safety of GNSS positioning if included in future certification standards.

This paper targets the robust detection of pseudorange measurements affected by NLOS signals. NLOS signals are signals received after one or several reflections but without the reception of the direct ray. This means that they necessarily induce an additional delay in pseudo-range estimation. On the contrary to other GNSS error sources, such as atmospheric delays, satellite ephemeris, or clock errors, local threats are difficult to predict and to model due to their dependency on the environment and time, and remains a major issue for safe and accurate GNSS-based positioning solutions in urban environments. The state-of-the-art algorithms largely include NLOS detection and mitigation. Solutions are proposed at the different stages of the receiver chain: from the antenna design (Suzuki et al., 2020b) to PVT estimator (Bressler et al., 2016), or by redundancy with other sensors such as inertial measurement units (Crespillo et al., 2018), LiDARs (Wen et al., 2019), or cameras (Marais et al., 2014). These classical approaches for handling NLOS in some cases may require additional sensors, expensive equipment, or specific detection methodology highly dependent on satellite geometry and current signal situations, whose specific performance is difficult to model. Given the inherent complex nature of signal propagation and receiver signal processing in urban scenarios, the detection of NLOS signals is a perfect use case for artificial intelligence algorithms and, in particular, machine learning (ML). The presence and impact of this threat depends on multiple factors that cannot be correctly considered by classical parametric tools to ensure conservative position estimation. The application of ML for GNSS positioning and, in particular, for NLOS detection represents, however, different challenges so that the methodologies satisfy generalization.

This paper first provides a literature research study on the use of artificial intelligence algorithms in the GNSS domain, focusing on the detection of local GNSS threats like NLOS. In particular, the main advantages, limitations, and specific algorithms used are discussed and summarized. Then, a new methodology is proposed to normalize the features with respect to the specific antenna installation and receiver. This generalizes the applicability of an already trained algorithm to a different hardware setup. A new logistic regression algorithm is proposed that handles features from multiple frequencies based on the creation of multiple estimation branches. Finally, NLOS detection results are shown for the normalization, training, and validation of the proposed algorithms in comparison with other state-of-the-art approaches. The evaluations are based on several hours of static data collected both in open-sky scenarios and two different locations with nearby buildings.

## 2 Use of machine learning and artificial intelligence for satellite navigation

### 2.1 Previous work

The use of artificial intelligence (AI), in particular ML algorithms for GNSS positioning, has increased in the last few years. Their capacity to model complex phenomena and relationships between parameters is very promising for multipath and NLOS detection, characterization, and mitigation in urban areas. First papers with ML addressed the use of classifiers for LOS/NLOS distinction such as a binary decision tree (Yozevitch et al., 2016) or an adaptive neuro fuzzy inference system (ANFIS) (Sun et al., 2019a). Then, ML-related publications focused on multipath and NLOS detection for mitigation (Hsu, 2017a; Suzuki et al., 2017) with or without a distinction between the two states of reception. Xia et al. (2020), for example, considered any anomaly, including NLOS reception. Orabi et al. (2020) developed a novel NN-based DLL (NNDLL) to mitigate multipath errors by focusing on the autocorrelation function computed in the receiver. Some other papers address the problem at the pseudorange error level, developing pseudorange error models. Sun et al. (2019b) used a gradient boosting decision tree (GBDT)-based method to predict the pseudorange errors by considering the signal strength, satellite elevation angle, and pseudorange residuals. For most of these studies, the benefits are evaluated by quantifying how this knowledge helped increase the position accuracy. Thanks to SDR, signal processing can be accessed and used for NLOS multipath detection (Suzuki et al., 2020a). Finally, some other applications of ML based on GNSS measurements are mentioned: for context detection (Gao and Groves, 2020), spoofing detection (Silvio Semanjski and Muls, 2020), the detection of GNSS ionospheric scintillation (Linty et al., 2018), or based on signals such as the Jammer Classification of Ferre et al. (2019) that are out of the scope of our study.

### 2.2 Challenges

As ML relies on data, the first step of work is to determine the features to be exploited, as well as the technique. As introduced previously, most of the studies rely on GNSS observables. The carrier-to-noise ratio (C/N0) is the most popular feature used for LOS/NLOS classification but has shown its inefficiency if used alone due to overlay between LOS and NLOS distributions of C/N0 (Wang et al., 2015). In 2016, Yozevitch et al. (2016) considered adding pseudorange residuals and satellite elevation to be combined, on one hand, in a decision tree (DT) for supervised classification and, on the other hand, expectation maximization (EM) as unsupervised classification. The authors have shown that both proposed algorithms outperformed the classical binary C/N0 thresholding method.

Recent investigations explore the use of support vector machine (SVM) such as in Xu et al (2020). New features from the smartphone-level GNSS chip are added to C/N0: satellite elevation, pseudorange (PR), and pseudorange rate consistency (PRC) that provides consistency between the pseudorange rate from pseudorange measurements and the Doppler shift. Hsu (2017b) also relied on SVM but detected LOS/NLOS signals and multipath, adding the difference between two consecutive errors of the carrier-to-noise ratio (Δ C/N0) as a feature. In these techniques, the PR is obtained after the position computation by subtracting the estimated range and all the known terms. In case the position estimation was conducted perfectly, the PR would equal the value of noise and multipath, while the PRC is the difference between the changing rate of the pseudorange measurements and the Doppler shift. Sun et al. (2019b) also considered LOS/NLOS signals and the multipath. This is conducted by using C/N0, satellite elevation, and PR, while the ML technique used is the DT. Furthermore, Tomohiro and Nobuaki (2022) used the SVM ML technique with unique features calculated by using receiver-independent exchange format-based information and pseudorange residual checks.

One of the main restrictions of Yozevitch et al (2016), Xu et al (2020), and Sun et al (2019b) is that the authors assume that low-elevation satellites always imply NLOS signals. Although low-elevation satellites are more likely to cause NLOS signals, this is not always the case. Moreover, the decision of an algorithm using the low-elevation satellite/NLOS signal feature is going to be restricted and conditioned by the elevation of the obstacles present in the training dataset.

Another notable restriction that occurs in Yozevitch et al. (2016), Xu et al. (2020), Sun et al. (2019b), Hsu (2017a), and Tomohiro and Nobuaki (2022) is their use of the pseudorange residuals. As it has been mentioned previously, PR is computed only after the position computation, which makes it dependent on the algorithm used for positioning, and consequently, the detector is not valid for different estimators. Moreover, position estimation is directly affected by the number of satellites (alongside other satellite properties) that are available for position estimation, which would cause the PR to vary for each situation. This makes the detection of LOS/NLOS a function of not only the satellite on review but also the rest of the satellites as well.

Furthermore, another limitation to C/N0-based methods, as explained in Yozevitch et al. (2016), is that C/N0 is strongly dependent on the installation used for data recording. In other words, the results are degraded when the installation used for training differs from the later installation. Lastly, a shortcoming of the state-of-the-art approach is the use of single-frequency features. Exploitation of multiple frequencies can enhance the NLOS detection capability. One of the reasons that the literature is limited to single frequency may be the difficulty to handle the intermittent presence of secondary frequencies like L2 or L5/E5 in the training and validation process.

As the choice of the relevant features, the choice of the ML algorithm is an important component of the proposals. Xu et al. (2018) compared the SVM method with other ML methods such as k-nearest neighbors (KNN), neural network (NN), and DT and concluded that SVM offers the best performance with commercial GNSS and decent generalization ability. Most recent papers explored more complex ML-based processes. Zhang et al. (2021) investigated how the combination of a fully connected neural network (FCNN) and long–short-term memory (LSTM) network not only allow in predicting satellite visibility but also PR error based on GNSS measurements. The interest of LSTM is the extraction of context information from sequential GNSS measurements. Sun and Fu (2022) proposed a stacking ensemble learning (SEL) method for the NLOS detection of GNSS. It also consists of two levels of ML models. The goal is to combine the views of different models to the measurement features to address the shortcomings of each single model and improve the model’s generalization.

Based on signals and thanks to SDR, Li et al. (2020) and Munin et al. (2020) show how one can detect the multipath contamination (not only NLOS) on the correlator output signal based on the deep neural network (DNN) for the former and the conventional neural network (CNN) converting the incoming signals into images at the time and frequency domains for the latter. These different methods and associated features are summarized in Table 1.

**TABLE 1:** S, supervised; US, unsupervised; DT, decision tree; EM, expectation maximization; SVM, support vector machine; DNN, deep neural network; CNN, convolutional neural network; FCNN, fully connected neural network; LSTM, long short-term memory; N, number of received satellites; (N)PR, (normalized) pseudorange residual; PRP, pseudorange residual percentage; PRC, pseudorange consistency; EL, elevation; AZ, azimuth; PDOP, precision of dilution; SFM, SNR fluctuation magnitude; ACF, autocorrelation function.

Purpose	Method	Technique	Feature vector	Reference
LOS/NLOS Classification.	S	DT	[C/N0 L1, EL, PR]	Yozevitch et al. (2016)
	US	EM	[C/N0 L1, EL, PR]	Yozevitch et al. (2016)
	S	SVM	[C/N0 L1, EL, NPR, PRC]	Xu et al. (2020)
	S	SVM	[C/N0 L1, ΔC/NO, PR, PRC]	Hsu (2017b)
	S	SVM	[C/N0, EL, NPR, PRC, SFM]	Tomohiro and Nobuaki (2022)
	S	DT	[C/N0 L1, EL, PR]	Sun et al. (2019b)
	S	KNN, NN, SVM, and DT	[C/N0 L1, N, EL, PR, PRP, NPR]	Xu et al. (2018)
Multipath detection	S	SVM	[C/N0 L1, ΔC/NO, PR, PRC]	Hsu (2017a)
	S	DT	[C/N0 L1, EL, PR]	Sun et al. (2019b)
	S	NNDLL	[ACF]	Orabi et al. (2020)
	S	DNN	[PRN code, I/Q Samples]	Li et al. (2020)
	S	CNN	[ Image of Signal in Time and Frequency]	Munin et al. (2020)
Signal anomaly detection	S	Clustering	[C/N0 L1, PR, PDOP, N, PRC]	Xia et al. (2020)
Scintillation	S	DT	[ $\bar{SI}$ , *σ*_*SI*_, *cov*(*I* ^2^, *Q* ^2^)]	Imam and Dovis (2020)
Code error estimation	S	FCNN + LSTM	[C/N0 L1, EL, AZ, PR, PR^2^]	Zhang et al. (2021)

### 2.3 Current limitations

If ML and deep learning are promising tools to model complex phenomena as the GNSS local effects in urban areas, the state-of-the-art algorithm is quite recent and still needs to be completed with deeper analyses and better accuracy of the models.

With respect to the selection of features for the ML/AI algorithms, we see the possible limitations and challenges of the state-of-the-art algorithms:

• Elevation: Using elevation as a feature may bias the model with respect to the training data. For instance, if training data were obtained in scenarios where buildings are predominately of a certain altitude, the model may not represent well other circumstances with lower or taller buildings that block the LOS signal at different elevations. If the training data consist of data covering in many and different scenarios (quite challenging in practice), then the elevation, as a feature, may not include such important information. Therefore, including elevation as a feature should be treated with care or completely avoided.
• Number of visible satellites: Although the limited visibility of the satellite may be the indication of being in an urban canyon, similar as with the elevation case, a model trained with data predominantly in given scenarios may bias the model for a general situation.
• Pseudorange PVT residuals: This information is a result of the PVT estimation process, which depends on many different factors like the number of visible satellites, number of constellations used, current geometry, and the specific error model used for each of the satellite measurements. Therefore, it may be very difficult to cover with enough training data in every possible situation of residuals. This may lead again to biased model estimation in many scenarios different from those that the training data represented.
• C/N0: Specific behavior of C/N0 is dependent on a specific satellite or constellation transmitted power, specific antenna gain diagram and spatial response, and a specific GNSS receiver C/N0 estimation method. Using a trained model with a specific setup and satellite data may not be properly extrapolated to other frequencies, constellations, antenna installation, or specific receivers.
• Training dataset availability: The limit of supervised learning algorithms is the availability of labeled datasets for training or their building. Indeed, the knowledge with certainty of the satellite state of reception LOS or NLOS still remains an issue (Xia et al., 2020), in particular in kinematic measurement campaigns. In the literature, some datasets are labeled, thanks to 3D models and comparison of the satellite positions with the model. It requires the accurate knowledge of the user position inside the model. Some others rely on fish-eye images of the surroundings. This requires a good calibration of the fish-eye lens and synchronized GNSS/image acquisition if used in a kinematic mode. A specific attention highlighted by Xu et al. (2020) concerns the classification of satellites located at the boundaries between masks and the sky. As an alternative, Xia et al. (2020) implemented an unsupervised clustering-based anomaly detection technique (HDBSCAN) that intends to detect and classify NLOS (but also other anomalous errors) and construct labeled offline training. Unsupervised methods solve this issue, but in the methods tested by Yozevitch et al. (2016), the unsupervised EM method shows much larger NLOS error rates than the supervised DT.
• Generalization ability is another challenge. Indeed, as the NLOS effects are directly dependent on the close surroundings of the receiver, one needs to ensure that the training dataset can represent the different cases and configurations that will be encountered while using the model (Xia et al., 2020).

Independently of the feature choice, some other issues need to be considered in the following. A rigorous analysis of the performance of such algorithms is still difficult for LOS/NLOS detection because of the difficulty to have a ground truth, and for the deep learning algorithms, it is difficult because of hidden layers. Lastly, in all these satellite states of reception prediction or measurement predictions, the transitions over time are neglected and do not consider the filtering techniques used by the receiver that will introduce latency on the GNSS feature variation.

## 3 Non-line-of-sight (NLOS) signals

A LOS signal has a direct geometric line of sight between the satellite and the receiver, while NLOS signals occur when the LOS between the satellite and the receiver is partially or entirely obstructed by one or multiple objects. However, despite the obstruction, some reflected signals still reach and are tracked by the receiver. Since the carrier frequency of NLOS GNSS signals experiences an additional attenuation due to the reflections, the carrier-to-noise power ratio metric is typically also affected by this effect.

**FIGURE 1:**
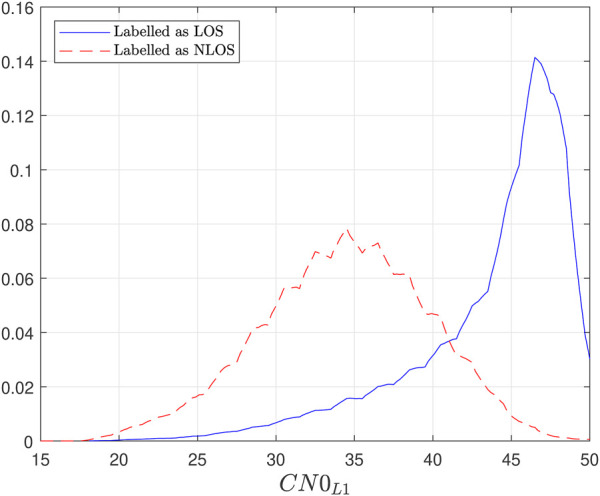
PDF of *C*/*N*0_*L*1_ for LOS and NLOS signals.

However, one difficulty when using C/N0 for signal classification is that a strong GNSS signal is a very likely indicator of a LOS signal, while a week signal is not exclusively an indicator of a NLOS signal. The reception of weak signals can be caused by a variety of other factors such as longer travel distance of the signal through the atmosphere (typically at lower elevation angles), antenna specific gain diagram, installation and specific placement, or the presence of interference. Additionally, a NLOS signal can be received under certain conditions, without being largely attenuated. As an example, Figure 1 shows a typical estimated distribution of C/N0 in the GPS L1 frequency under LOS and NLOS conditions. It can be observed that LOS signals have a probability distribution of higher values, while the probability distribution of NLOS signals is more focused on the possible C/N0 values. Moreover, one can see the overlap between the two distributions for a large range of C/N0 values.

## 4 Branched machine learning-based NLOS detection methodology

**FIGURE 2:**
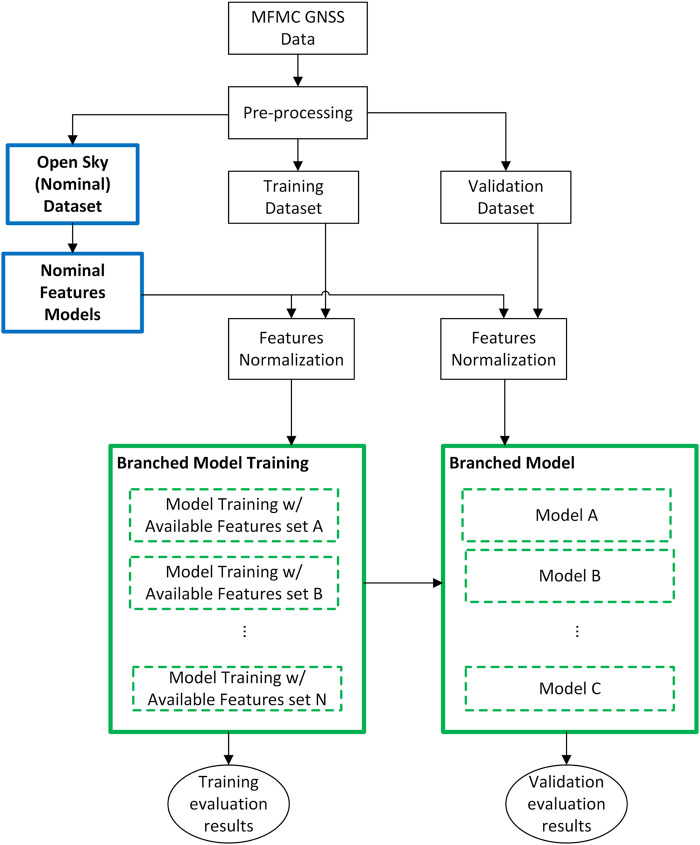
Block diagram of the pre-normalized and branched machine learning algorithm.

This paper proposes a methodology for the application of ML algorithms for GNSS measurement processing that targets its generalization and exploitation of intermittent GNSS measurement in certain frequencies in challenging environments. Figure 2 shows a general diagram considering the following main elements:

1. The introduction of an off-line modeling step to characterize the performance of features in open-sky (nominal) scenarios that is used as a reference model for feature normalization.
2. The typical ML training and validation/testing step.
3. The pre-normalization of features before its use in the ML algorithm.
4. The design of branched or parallel ML algorithms in training and validation to handle different situations of measurement availability in certain frequencies.

In the following sections, the details about feature selection, pre-normalization, and the selected logistic regression algorithm for GNSS NLOS detection are introduced.

### 4.1 Feature selection

Based on the literature review in Section 2, this section presents the selected features for this work.

#### 4.1.1 Carrier-to-noise ratio (C/N0)

C/N0 is expected to be one of the main descriptors for the LOS/NLOS prediction. The use of C/N0 in multiple bands is included as features. In particular, for GPS, L1, L2, and L5 have been taken into account, while for Galileo, E1, E5a, and E5b. The levels of C/N0 are dependent, on one hand, on the elevation of the satellite since a lower elevation implies a larger distance traveled through the atmosphere from the satellite to the receiver. On the other hand, the antenna gain and immediate installation setup plays a crucial role in the signal attenuation and multipath (Kliman and Crespillo, 2022), which affects the C/N0 level. Finally, the specific receiver tracking loop configuration may also be a factor. In order to generalize the designed ML algorithms, a pre-normalization of C/N0 is, therefore, introduced in order to decouple the ML algorithm from the specific antenna, installation, and receiver setup.

The normalization is performed assuming a Gaussian model distribution for each satellite elevation:

$${\bar{C/N0}}_{i,j}=\frac{C/N0_{i,j}-\mu_{j}\left(\theta_{i}\right)}{\sigma_{j}\left(\theta_{i}\right)},$$ (1)

where ${\bar{C/N0}}_{i,j}$ and *C*/*N*0_*i*,*j*_ are the normalized and measured C/N0 of the satellite *i* and frequency *j* measured in dB/Hz, respectively. *μ*_*j*_(*θ*_*i*_) and *σ*_*j*_(*θ*_*i*_) are the mean and standard deviation of the nominal reference Gaussian model for elevation *θ*, respectively. The reference nominal model parameters can be extracted from data recorded in an open-sky scenario. In this situation, GNSS threats like high multipath or NLOS signals are considered negligible so that it is possible to isolate the C/N0 level purely due to the installation.

#### 4.1.2 Pseudorange rate consistency (PRC)

Code and Doppler measurements are affected at different levels by NLOS. A modified version of the PRC defined in Xu et al. (2020) is, therefore, also included as a feature:

$$\bar{PRC}=\left|\frac{\Delta \rho }{\Delta t}-\left(-\lambda f_{D}\right)\right|,$$ (2)

where Δ*ρ* is the time difference of pseudorange measurement, Δ*t* is the time interval between measurements, *λ* is the wavelength of the signal frequency, and *f*_*D*_ is its Doppler frequency. Our primary goal in this paper is detecting the impact of NLOS on the primary constellation frequency (i.e., L1 for GPS and E1 for Galileo), and therefore, the PRC is computed based on the code and Doppler measurements of the corresponding frequency.

#### 4.1.3 Lock time

In different situations of signal re-acquisition after, for instance, exiting tunnels or a bridge, the receiver may first track a NLOS signal instead of the LOS signal. The lock time information *T*_*lock*_ is usually provided by receivers and may provide useful information about the probability of NLOS. Since the relevance of the lock time is at the beginning of signal tracking, and in order to avoid overfitting, the lock time is considered to be a feature in the following way:

$$T_{\mathrm{lock}}^{\prime }=\left\{\begin{array}{cc} T_{\mathrm{lock}},\; & \text{if }\;T_{\mathrm{lock}}<T_{\text{min}}.\\ T_{\text{min}},\; & \text{otherwise}. \end{array}\right.$$ (3)

The threshold value *T*_min_ = 15 *s* has been set heuristically in this work, and it is expected to play a bigger role in future work in dynamic scenarios. As for the case of PRC, we considered the lock time of the primary constellation frequency (i.e., L1/E1) since it has been observed that it is the frequency that is typically first tracked by receivers.

#### 4.1.4 Complete feature vector

The signals and measurements from different constellations have to be considered separately since each constellation provides information on different frequencies. For GPS and Galileo, the following feature vector is considered:

$$x_{\text{GPS}}=\left[{\bar{C/N0}}_{L1},{\bar{C/N0}}_{\text{L}2},{\bar{C/N0}}_{\text{L}5},{\bar{PRC}}_{L1},T_{\mathrm{lock,L1}}^{\prime }\right]$$ (4)

and

$$x_{\text{GAL}}=\left[{\bar{C/N0}}_{E1},{\bar{C/N0}}_{\mathrm{E5a}},{\bar{C/N0}}_{\mathrm{E5b}},{\bar{PRC}}_{E1},T_{\mathrm{lock,E1}}^{\prime }\right].$$ (5)

### 4.2 Branched logistic regression

The main goal of this work is to classify a satellite measurement as LOS or NLOS. Depending on how this information is later used in a positioning algorithm, it may also be important to know the likelihood of the classification result. For the users to have the flexibility to exclude a NLOS signal or include this measurement with a lower weight, we have chosen a logistic regression algorithm for this work. The logistic regression uses a sigmoid mapping function to estimate a probability of the target classification of LOS/NLOS signals. The probability of receiving a LOS signal is mathematically expressed as

$$\mathrm{Pr}\left(x;\beta \right)=\frac{1}{1+e^{-\beta^{T}x}},$$ (6)

where ***x*** is the vector of features used as input and ***β*** is the vector of coefficients that must be fitted.

If a final binary classification is desired, the criteria Pr(***x***) < 0.5 can be used to determine NLOS reception and Pr(***x***) ≥ 0.5 for the LOS reception. The training of the algorithm is usually conducted by fitting the parameters ***β*** with the following mean square error cost function:

$$J\left(\beta \right)=\frac{1}{M}\overset{M}{\underset{i=1}{\sum }}{\left(\mathrm{Pr}\left(x_{i};\beta \right)-y_{i}\right)}^{2},$$ (7)

where M is the size of the training dataset and *y*_*i*_ is the label of the data. The typical approach to tackle this optimization problem is to use a gradient descent algorithm, as in Mitchell (1997).

In challenging GNSS scenarios where the reception of NLOS signals is expected, the tracking of the GNSS signals is stressed by multiple possible effects like multipath and intermittent shadowing. In this situation, it is common for the receiver to only be able to provide measurements in some of the received signal frequencies. It is found in general that the tracking of L1/E1 signals (and therefore the computation of code and carrier-phase measurements) is normally the most available, while the tracking of L2 and L5/E5 suffers for intermittent availability in urban scenarios. The possible presence or absence of certain measurements (i.e., features) at different moments challenges the design, training, and validation of ML algorithms that normally need a fixed number of features. On the other hand, the fact that certain frequency channels cannot be tracked can contain by itself important information about the expected reception of NLOS signals on the main channel L1/E1. In this work, in order to be able to exploit the C/N0 measurements from all available frequencies and to perform the LOS/NLOS as well in cases where partial frequency measurements are available, we proposed a branched model training and validation methodology. For this, we consider the training of different models with a different number of features from Eq. 4 and Eq. 5, which we called here branched logistic regression. In particular, we consider, in total, five different models for the GPS and Galileo constellation with the following number of features:

• Model A: $x_{\text{GPS,A}}=\left[{\bar{C/N0}}_{L1},{\bar{C/N0}}_{\text{L}2},{\bar{C/N0}}_{\text{L}5},{\bar{PRC}}_{L1},T_{\mathrm{lock,L1}}^{\prime }\right]$ .
• Model B: $x_{\text{GPS,B}}=\left[{\bar{C/N0}}_{L1},{\bar{C/N0}}_{\text{L}2},{\bar{PRC}}_{L1},T_{\mathrm{lock,L1}}^{\prime }\right]$ .
• Model C: $x_{\text{GPS,C}}=\left[{\bar{C/N0}}_{L1},{\bar{C/N0}}_{\text{L}5},{\bar{PRC}}_{L1},T_{\mathrm{lock,L1}}^{\prime }\right]$ .
• Model D: $x_{\text{GAL,D}}=\left[{\bar{C/N0}}_{E1},{\bar{C/N0}}_{\mathrm{E5a}},{\bar{C/N0}}_{\mathrm{E5b}},{\bar{PRC}}_{E1},T_{\mathrm{lock,E1}}^{\prime }\right]$ .
• Model E: $x_{\text{GAL,E}}=\left[{\bar{C/N0}}_{E1},{\bar{PRC}}_{E1},T_{\mathrm{lock,E1}}^{\prime }\right]$ .

For the validation (and real application) of the proposed approach, only one model per constellation is used at a time. This would be the one that uses features from more frequencies depending on the available data provided by the GNSS receiver in a given epoch.

### 4.3 Labeling methodology

In order to train and validate the proposed branched logistic regression solution, the determination of true LOS or NLOS satellite signals is determined by discrimination based on horizon information. The process considers, first, the determination of open-sky horizon determination from the antenna point of view. For each of the satellite measurements provided by the receiver, the elevation and azimuth of the respective satellite are computed. Finally, the elevation and azimuth are compared with the previously determined horizon to determine whether a satellite is in direct LOS or not. In Section 5.2, more details about the experimental horizon determination process used in this work are provided.

## 5 Experimental methodology

### 5.1 Measurement setup and campaign

The training and validation of the algorithm in this work was performed with measurements collected with a DLR-designed antenna and *Septentrio mosaic-X5* receiver.

**FIGURE 3:**
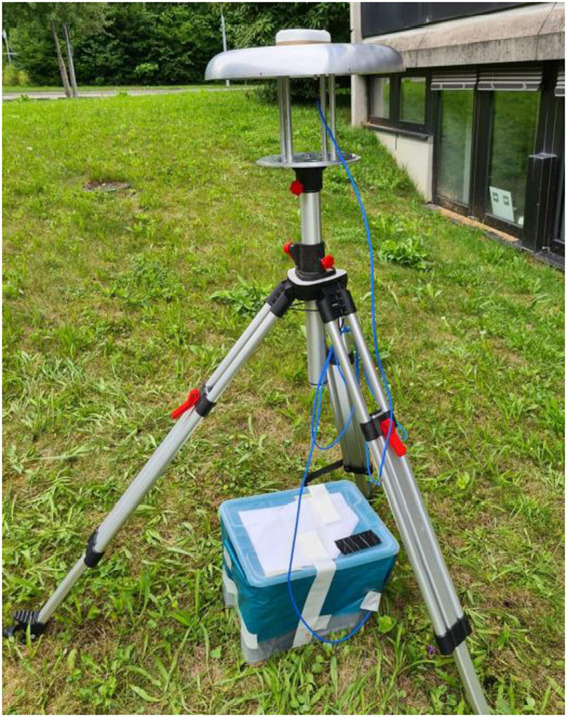
Antenna installation.

**FIGURE 4:**
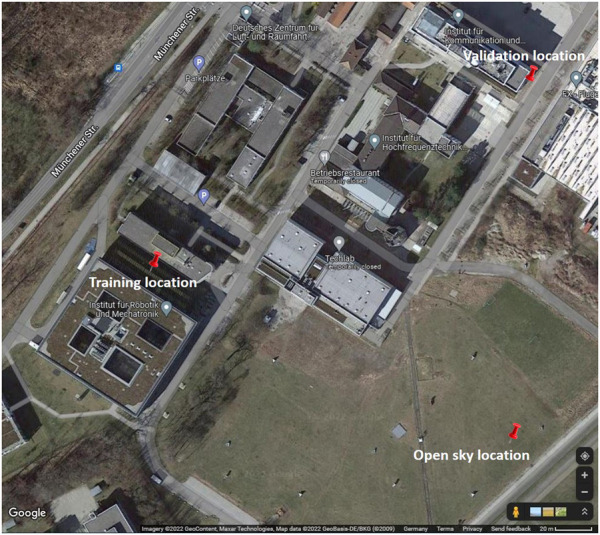
Locations of the open-sky location, training location, and validation location.

The antenna was placed on a tripod, while the receiver and power source were placed in a plastic box to be protected from weather conditions, as shown in Figure 3. The measurement campaign took place for several days and in a total of three measuring locations. In order to ensure a full repeating orbit cycle (of at least the GPS constellation), more than 24 consecutive hours were recorded at every location. The sampling rate was set to 10 Hz. All locations used were within the premises of the DLR facility, as shown in Figure 4.

The first dataset was collected in an open-sky environment to support the derivation of the nominal reference C/N0 models (on Figure 4 labeled as “Open sky location”). The location was chosen for its good visibility of satellites for all elevation and azimuth calculations. Some pre-screening has been applied as the data preparation pre-processing step to avoid low-elevation effects.

**FIGURE 5:**
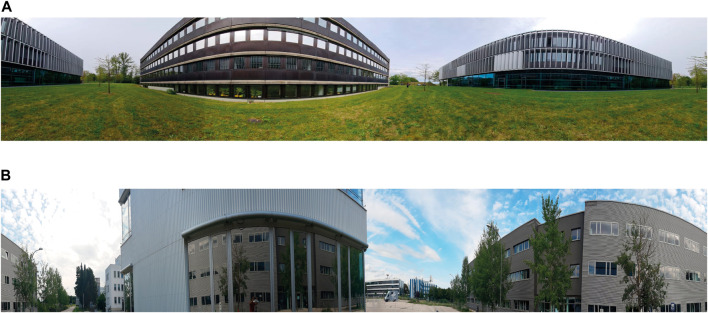
**(A)** Panoramic view of the training location. **(B)** Panoramic view of the validation location.

The two other datasets were collected under locations chosen because of the challenging scenarios with surrounding buildings that blocked parts of the sky visibility (on Figure 4 labeled as “Training location” and “Validation location”). The panoramic view from the standpoint of the receiver for both the training and validation locations is shown in Figure 5.

The following Table 2 gives an overview of the used datasets. The locations are used to collect them, the usage, and their total length.

**TABLE 2:** Description of collected datasets.

Dataset	Location	Usage of the dataset	Length
Dataset 1	Open-sky location	Nominal model + validation of normalization	22 h
Dataset 2	Training location	Training of the model	87 h
Dataset 3	Validation location	Validation of the model	46 h

### 5.2 Horizon determination

The horizon determination for the training and validation locations is the first step to determine the LOS/NLOS label of the signal. The horizon was determined considering the angle and the distance to the upmost edge of every obstacle on the horizon around the measuring location. The buildings shape allows for them to be modeled with only the angle and the distance to their corners, as well as the length of the building itself. The location of the antenna was determined in post-processing with multi-pass Kalman filtering.

**FIGURE 6:**
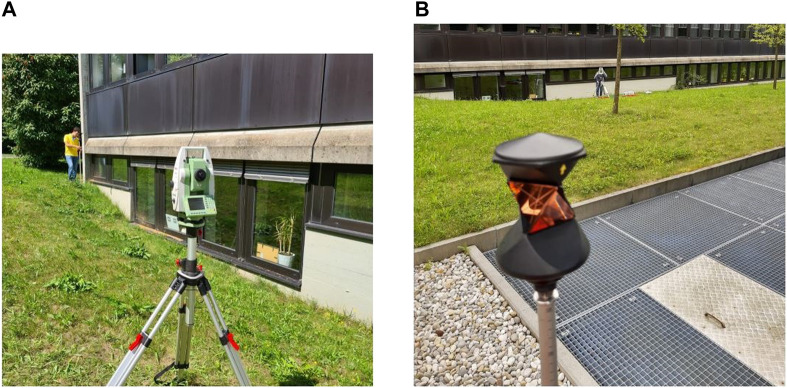
**(A)** Leica total station and **(B)** 360° prism.

In Yozevitch et al. (2016), a mobile application was used for determining the horizon, while, in this paper, we opted for a geodetic total station (TS), with a higher expected accuracy. A total station is an instrument that uses an electronic optical distance meter for determining distances and an electronic angle meter for measuring horizontal and vertical angles. The total station used is a *Leica TPS1200* alongside a 360° prism, as shown in Figure 6.

**FIGURE 7:**
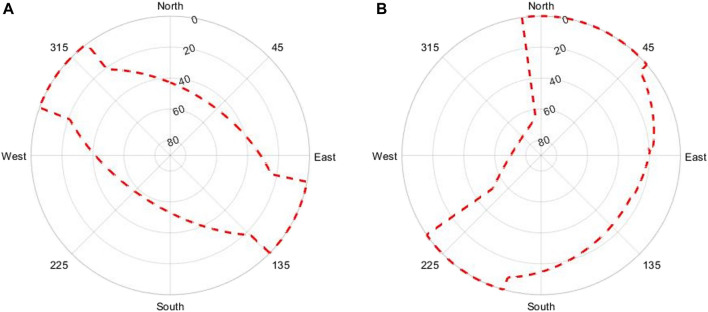
**(A)** Horizon training location. **(B)** Horizon validation location.

When using a 360° prism, the angles are determined with an accuracy of 1, while the distances, with an accuracy of 2 mm + 2 ppm. In addition, the accuracy of the ground truth is not relevant when using TS since a local coordinate system was set up and all distances and angles were determined relatively with respect to the measurement location. The obtained sky plots of the training and validation locations can be seen in Figure 7.

## 6 Results

This section provides information about the labeling and normalization data results and the performance of the NLOS detector in comparison with other state-of-the-art algorithms. The detector is ultimately a classification algorithm whose performance is evaluated by means of a confusion matrix where the accuracy of true(t)-false(f) and positive(p)-negative(n) classifications is computed normalized with respect to the label category. Note that in terms of the confusion matrix, a positive classification is defined as the classification of a signal as LOS. The overall accuracy is defined as

$$\text{Accuracy}=\frac{tp+tn}{tp+fp+tn+fn}.$$ (8)

### 6.1 Pre-normalization evaluation

**FIGURE 8:**
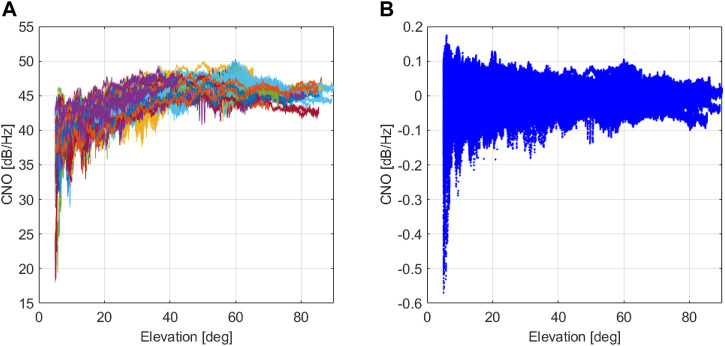
Normalization of the C/N0 feature (example for GPS L1). **(A)** Nominal open-sky C/N0 data. **(B)** Normalized open-sky C/N0 feature.

Figure 8 (left) shows the recorded C/N0 for the GPS L1 band in the open-sky location. From these data, the reference model parameters *μ*_*L*1_(*θ*) and *σ*_*L*1_(*θ*) were computed for each elevation. Figure 8 (right) presents the same data after the normalization process. The dependency with the elevation and the installation has been clearly reduced. This justifies the necessity of normalizing C/N0 in order to avoid a high rate of false alarm at low elevation.

**FIGURE 9:**
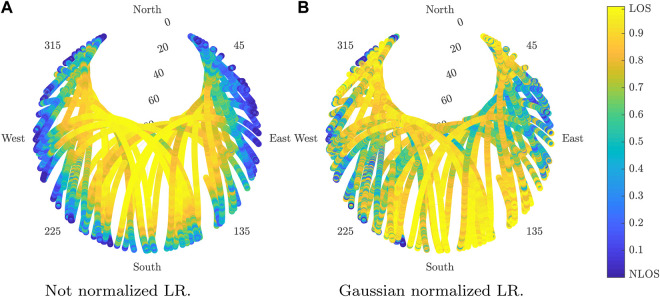
Estimated LOS probability using LR in open sky for GPS. **(A)** Not normalized LR. **(B)** Gaussian-normalized LR.

In order to highlight the importance of normalization, we compare the detector with and without normalization in an open-sky scenario where the LOS probability should be always *p*_*LOS*_ > 0.5. Table 3 exhibits the confusion matrix for GPS in this scenario whereas Figure 9 shows its respective skyplot. As expected, the not normalized version considers NLOS satellites as those with low elevation. This is caused by the reduction of C/N0 just because of the elevation. As shown numerically in Table 3, the normalized branched LR clearly outperforms the not normalized LR. The difference in terms of accuracy could potentially be even higher, but in practice, it is difficult that the scenario for recording the data is perfectly open sky.

**TABLE 3:** Confusion matrix of the detector in open sky for GPS.

Label	Without pre-normalization		With pre-normalization	
	LOS	NLOS	LOS	NLOS
LOS	74%	26%	91%	9%
NLOS	-	-	-	-
Accuracy	74.2%		90.7%	

### 6.2 Training and validation evaluations

In order to make a comparison between the state-of-the-art algorithm and the design presented in this work, we have trained a detector according to the algorithms and features that can be found in the literature. In particular, the most used ML algorithms in the state-of-the-art algorithm are the DT and the linear SVM, as shown in Table 1, and they are, here, used as a reference for comparison. These algorithms do not consider either the normalization step or the use of measurements from multiple frequencies. Therefore, an adapted model and feature vector have been used for these algorithms. Details about the design of the chosen state-of-the-art algorithms can be found in Supplementary Appendix A.

Table 4 shows the performance of the DT and SVM algorithms over the training dataset for GPS and Galileo, respectively. The DT is slightly better than the SVM in terms of accuracy. However, the SVM has a more balanced distribution of error. This is more noticeable for the case of GPS.

**TABLE 4:** GPS and Galileo confusion matrix results for state-of-the-art algorithms.

Label	GPS				Galileo			
	SVM detector		DT detector		SVM detector		DT detector	
	LOS	NLOS	LOS	NLOS	LOS	NLOS	LOS	NLOS
LOS	76%	24%	83%	17%	86%	14%	76%	24%
NLOS	14%	86%	21%	79%	21%	79%	17%	83%
Accuracy	80.8%		82.1%		80.6%		81.2%	

Table 5 presents the confusion matrices for GPS and Galileo for the proposed algorithm in this work for the training and validation datasets. The results show that the inclusion of C/N0 from additional bands clearly outperforms the state-of-the-art algorithm results in Table 4.

**TABLE 5:** Confusion matrix of the proposed detector with training and validation datasets.

Label	Training				Validation			
	GPS		Galileo		GPS		Galileo	
	LOS	NLOS	LOS	NLOS	LOS	NLOS	LOS	NLOS
LOS	89%	11%	91%	9%	88%	12%	92%	8%
NLOS	14%	86%	14%	86%	20%	80%	10%	90%
Accuracy	87.6%		88.9%		84%		91.4%	

**FIGURE 10:**
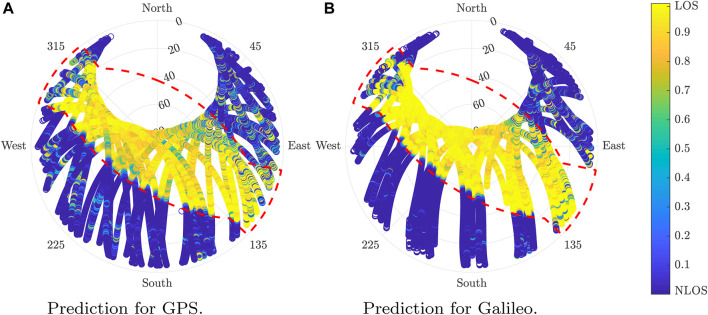
Estimated LOS probability in the training location for GPS and Galileo. **(A)** Prediction for GPS. **(B)** Prediction for Galileo.

Figure 10 provides further insight about the performance of the LOS probability determination by showing the skyplot results in the training scenario.

**FIGURE 11:**
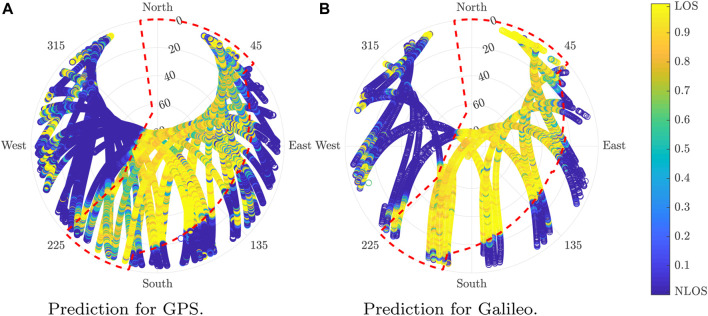
Estimated LOS probability in the validation location for GPS and Galileo. **(A)** Prediction for GPS. **(B)** Prediction for Galileo.

Figure 11 illustrates the skyplot results of the LOS probability determination of the predictions for GPS and Galileo over the validation dataset.

The algorithm outperforms in the state-of-the-art algorithm. The accuracy obtained over the validation scenario is lower than the scenario used to train the model. Although this is typically the normal situation with ML algorithms, it could also be caused by additional factors such as a higher error labeling, the data due to the difficulties for modeling, and the validation scenarios. Despite the efforts made in the location choice, some complex elements to model such as trees remained were not taken into account for the horizon determination.

Finally, we show the classification results over two C/N0 features of interest to get an insight about the regions in which the classification considers LOS or NLOS. We consider Eq. 6 with the threshold probability of the classification on the left-hand side of *p*_*LOS*_ = 0.5. Under that restriction, we solve Eq. 6 leading

$$\beta^{T}x=0.$$ (9)

**FIGURE 12:**
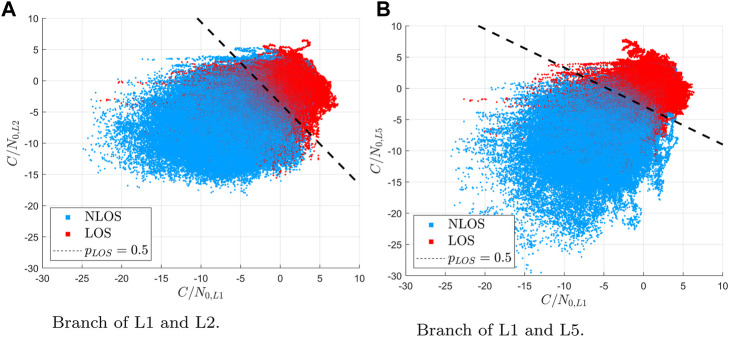
Decision areas for different branches of the model for GPS. **(A)** Branch of L1 and L2. **(B)** Branch of L1 and L5.

Eq. 9 represents a hyper-surface with respect to the input features (over training dataset) given in the model parameters ***β***. We fix all the input features to the mean values from the datasets except for the two features of interest. Figure 12 depicts the point cloud of two C/N0 features along with the detection regions separated by a black line of probability of 0.5. In particular, Figure 12A shows the features of the branch tracking only L1 and L2 (Model B), while Figure 12B considers the case of only L1 and L5 (Model C).

The figure shows how, despite some outliers, most of the points are correctly split into two regions. Moreover, observing the slant of the *p*_*LOS*_ = 0.5 line, it is appreciated that all *C*/*N*0 values are useful for differentiating between LOS and NLOS signals.

## 7 Conclusion

This paper presents a LOS/NLOS detector for GPS and Galileo that introduces two novel characteristics. First, the inclusion of multiple C/N0 from different bands in a branched scheme gives additional sustainable information that allows outperforming the current detectors in the literature to the best of the authors’ knowledge. Second, the normalization methodology proposed increases the robustness of the detector against changes in the installation or the location, as shown by the performance in the validation scenario. Additionally, the utilization of logistic regression admits the estimation of a LOS probability and not only the predicted label LOS or NLOS as it does to the DT and the SVM. This property, combined with the fact that the LR is more tractable mathematically than the DT and the SVM, allows obtaining some insights about the behavior of the detector. Moreover, the estimated LOS probability might provide helpful information for applications such as the position computation.

## Data Availability

The datasets presented in this article are not readily available because the datasets have been collected with proprietary equipment. Requests to access the datasets should be directed to omar.garciacrespillo@dlr.de.
