# Supplementary material for: Robust design of a machine learning-based GNSS NLOS detector with multi-frequency features
Source: Front Robot AI. 2023 Jul 28;10:1171255. doi: 10.3389/frobt.2023.1171255 (PMC10416230; doi:10.3389/frobt.2023.1171255)
Supplement: Supplementary file 1 [file RelatedArticle1.pdf]

APPENDIX: STATE-OF-THE-ART NLOS DETECTION IMPLEMENTATION

A Adapted Model Feature Vector

Algorithms based on decision trees and SVM for the LOS/NLOS classification proposed in the literature do not make use of measurement from multiple frequencies and do not consider a pre-normalization step. The feature vectors used for the GPS and Galileo model for those algorithms are respectively:

$$X'_{GPS} = [C/N0_{L1}, T'_{lock,L1}, \overline{PRC}_{L1}], \quad (1)$$

and

$$X'_{GAL} = [C/N0_{E1}, T'_{lock,E1}, \overline{PRC}_{E1}]. \quad (2)$$

B Decision Trees

The decision tree is a supervised algorithm for classification or regression. It is widely used because the final flowchart of the trained model can be visually analyzed. This allows extracting insights about the behavior of the algorithm.

As its name indicates, the structure of this algorithm is a tree where each leaf node represents a condition or decision rule. This decision rule is generally a threshold for a feature fitted as a function of the dataset. The more depth the tree has, the better the tree will be fitted. However, a very deep tree could cause overfitting to the training dataset losing generalization and, therefore, accuracy in the validation dataset.

The main advantages of decision trees are not only their visual representation but also their robustness against outliers. Moreover, decision trees give insights into which features are the most important. However, they can not seize all the features simultaneously, but instead, they prioritize only the main important ones. Another problem of this algorithm is its propensity to overfitting and the resulting unstable models where a slight variation in the input can entirely modify the output.

C Support Vector Machines

The SVM (Support Vector Machine) is a supervised algorithm which can work both for regression and classification. In contrast to the decision tree, this algorithm can consider a large number of features to obtain the best possible prediction. This is done by finding a hyperplane with the same dimensionality as the number of features that divides the two possible labels. Moreover, this can be extended to multiple classes by computing more hyperplanes.

Although the aforementioned hyperplane is the decision bound between both labels, multiple hyperplanes can be fitted with the same data by slight rotations or shifting. As this is not convenient, an additional criterial is included to find the optimal hyperplane. It consists in finding the hyperplane for which the distance with the closest points for each label must be maximal. An example for the case of two dimensions is shown in Fig. 1.

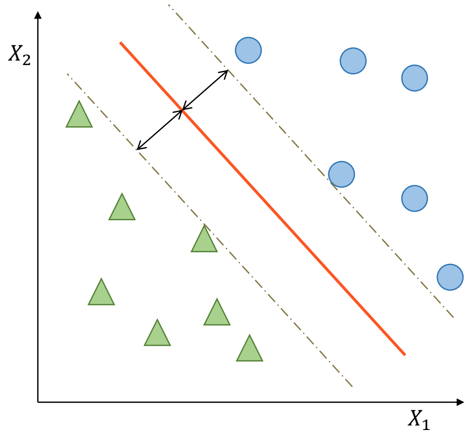

Figure 1. Example of SVM fitting for two dimensions.

If the most accurate bound is not linear, other shapes can be used as quadratic or radials by processing the features with the corresponding functions called kernels. However, computing complexity will increase with kernels different from the linear. In any case, even with the linear kernel, complexity is one of the main disadvantages of SVM and this restricts the amount of data that can be used for training.
